# Uncoupled Analysis of Stochastic Reaction Networks in Fluctuating Environments

**DOI:** 10.1371/journal.pcbi.1003942

**Published:** 2014-12-04

**Authors:** Christoph Zechner, Heinz Koeppl

**Affiliations:** 1 Department of Information Technology and Electrical Engineering, ETH Zurich, Zurich, Switzerland; 2 Department of Electrical Engineering and Information Technology, Technische Universität Darmstadt, Darmstadt, Germany; University of Pittsburgh, United States of America

## Abstract

The dynamics of stochastic reaction networks within cells are inevitably modulated by factors considered extrinsic to the network such as, for instance, the fluctuations in ribosome copy numbers for a gene regulatory network. While several recent studies demonstrate the importance of accounting for such extrinsic components, the resulting models are typically hard to analyze. In this work we develop a general mathematical framework that allows to uncouple the network from its dynamic environment by incorporating only the environment's effect onto the network into a new model. More technically, we show how such fluctuating extrinsic components (e.g., chemical species) can be marginalized in order to obtain this decoupled model. We derive its corresponding process- and master equations and show how stochastic simulations can be performed. Using several case studies, we demonstrate the significance of the approach.

## Author Summary

Cellular reaction networks show a substantial degree of complexity, and many of the regulatory mechanisms are yet to be discovered. Therefore, building detailed mathematical models of complete networks is not only unrewarding but most often impossible. Instead, focus is put on small subnetworks such as the transcriptional circuitry associated with a few genes. Practically, those subnetworks are still embedded in the cellular environment and are consequently modulated by it. The mathematical framework that we present in this work allows to build models of such subnetworks only, while still incorporating the impact of the environment. Such models are instrumental not only for advancing our biological understanding through them, but also for the reliable forward engineering of new subnetworks in synthetic biology.

## Introduction

Biochemical systems involving low-copy molecules demand for mathematical models that account for the intrinsic stochasticity [1]. In recent years, however, realization has grown that intrinsic noise alone cannot account for the observed substantial phenotypic variability among isogenic cells. That is, fluctuations in the intracellular environment, commonly termed extrinsic noise, represent an additional source of variability [2], [3], [4].

Several recent studies focus on separating intrinsic and extrinsic fluctuations through dual-reporter measurements [5], [6]. Other approaches model extrinsic noise through certain parameters (i.e., the translation rate) of a kinetic model which is calibrated subsequently using flow-cytometry [7], [8] or time-lapse microscopy data [9], [10]. All of those approaches have in common that they consider the biochemical process under study – i.e., the expression of a gene – as a small subpart that is embedded into a larger dynamical system. Accordingly, they rely on augmenting the original kinetic model by certain environmental components which are assumed to be fixed but random [11], [10], [8], [9] or fluctuating over time [5], [12], [13]. In fact, such models agree very well with the variability that is observed experimentally, but on their downside, suffer from the increased dimensionality - somehow defeating the original purpose of tractable dedicated models.

A natural question arising in that context is whether we can find a proper dynamical description of just the system of interest as if it was still embedded into its stochastically modulating environment. In other words, we aim to find a “self-contained” stochastic model that summarizes all system behaviors attainable under all possible realizations of the extrinsic fluctuations. Such models could then be used to perform an uncoupled analysis of a reaction network subject to extrinsic noise. The mathematical correct answer that we provide in this work is the marginalization of the system dynamics with respect to those extrinsic fluctuations. Interestingly it turns out that the resulting model exploits its own stochasticity to emulate the effect of extrinsic noise, leading to a self-exciting process. A simple instance of such self-excitation is the Polya urn scheme: at each draw from the Polya urn with balls of two colors the drawn ball and a fixed number of new balls of the same color as the draw are placed in the urn. This scheme is known to be equivalent to Bernoulli trials marginalized over random (and here correspondingly extrinsic) success rates [14]. Intuitively, due to its self-excitation, the number of draws of the same color for a Polya urn over repeated draws displays a much richer and dispersed dynamics than the number of successful draws in a Bernoulli trial with fixed success rate.

For the purpose of inference we recently proposed a first attempt of such marginalization for the special case of fixed but random environmental conditions [9]. In this work we develop a general mathematical framework from which the uncoupled dynamics can be constructed in a principled manner, regardless whether the environment is constant or dynamically changing.

## Results

### Mathematical framework

We describe the time-evolution of a stochastic reaction network by a continous-time Markov chain (CTMC) 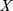 with 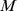 chemical species and 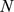 reaction channels. The system state at time 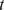 is denoted 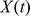 and we write its random path on time intervals 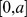 as 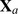. Throughout we will follow the usual convention to refer to upper-case and lower-case versions of a symbol as a random variable and its realization, respectively. Furthermore, we assume that 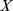 depends on another multivariate Markov process 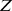 through its hazard functions in the form 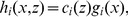
(1)with 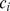 some positive function and 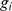 a polynomial determined by the law of mass-action, for instance. For reactions independent of 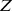, we thus have 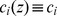. Typically, 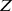 is another jump or diffusion process corresponding to a set of modulating *environmental* species or conditions that are considered extrinsic to the system of interest, whereas the species in 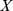 represent the actual system of interest. For example, 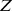 could be the fluctuating ribosome copy numbers affecting the kinetics of a gene regulatory network represented by 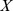. Although a more general treatment is possible, we assume a feed-forward structure between 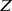 and 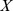, which means that 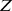 modulates 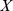 but not vice-versa. Consequently, the dynamics of the joint system 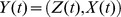 can be described by a Markov process 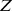 together with a conditional Markov chain 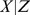.

#### Uncoupled dynamics

Mathematical descriptions of the joint system 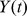 are readily obtained using available techniques for modeling Markovian dynamics [5], [15], [12]. For complexity reasons, however, we aim for models that can properly describe *only* the interesting components 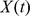. In order to see that marginalization over 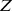 yields the desired model, let us first consider two dependent random variables 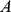 and 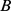 described by a joint probability distribution 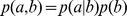. If we are interested in analyzing 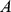 under all possible values of 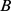, we need to average the probability at 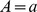 over all possible values of 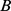, i.e., 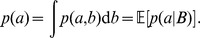



Note that as a consequence of averaging probabilities, any value of 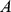 possible under the joint distribution 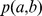 is possible under the marginal 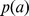, while this does not necessarily apply to 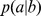 for any choice of 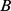. The resulting *marginal* distribution 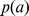 is an exact mathematical description of 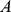 only, or in other words, it allows to analyze 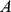 uncoupled from 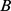.

**Figure 1:**
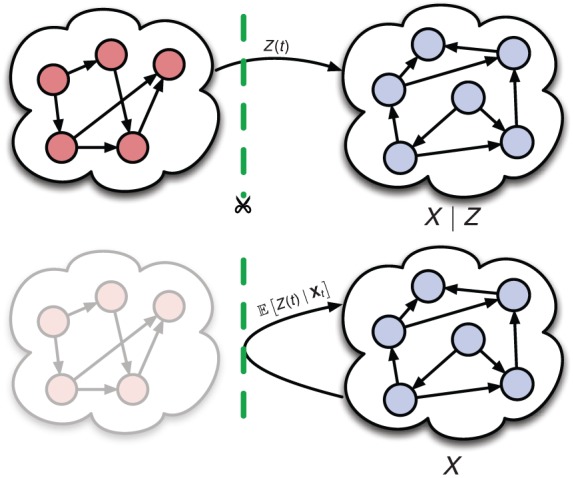
Uncoupled stochastic dynamics. The environmental process 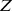 modulates the dynamics of the process under study 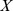, e.g., through one of its hazard functions. Marginalization with respect to 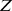 yields the uncoupled dynamics of 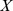, whereas the original dependency on the environment 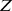 is replaced by its optimal estimator given the history of 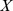. Consequently, the marginal process 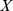 is self-exciting, i.e., it exerts a feedback on itself.

In case of the coupled processes 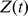 and 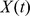, we analogously *marginalize* the joint Markov chain 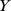 with respect to the environmental process 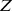. While such a marginalization involves several difficulties, the idea remains the same: we try to construct an uncoupled process 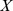 which directly admits the marginal path distribution 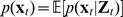. As a result, we obtain a jump process, which - in contrast to the conditional process 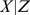 - no longer depends on the environment 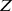. We remark that a straightforward marginalization of the joint master equation of 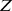 and 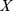 generally leads to intractable propensities [16], [5]. Based on the innovation theorem [17] we demonstrate in section S.1 in S1 Text that the hazard functions of the uncoupled process (later referred to as the *marginal* hazard functions) can be generally written as 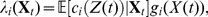
(2)where the expectation is taken with respect to the conditional distribution 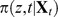. The latter describes the conditional probability of the environmental process 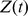 given the entire history (or filtration) of process 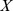 until time 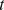. Using the expected value of that distribution, the feed-forward influence of 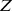 on the hazard functions of 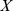 can be replaced by a deterministic function of 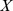, which no longer depends on the actual state of 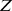. Instead, the uncoupled process 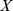 becomes *self-exciting*, meaning that it exerts a feedback on itself. Hence, given that we can evaluate Eq.2, we have a means to simulate 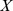 while bypassing the need to draw realizations of 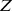. This has for instance been exploited for the exact simulation of diffusion-driven Poisson processes [18]. Note that the uncoupled process 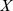 is no longer Markovian, since the conditional expectation - and hence the hazard function - possibly depends on the full process history 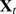. A schematic illustration of that uncoupling is given in Fig. 1.

#### Associated filtering problem

Although the construction of the uncoupled dynamics is general, any practical implementation thereof will depend on an explicit computation of the conditional expectation in Eq. 2. This expectation estimates the environmental state 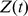 given the full history of the uncoupled process 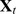 and therefore, can be understood as the solution to a *stochastic filtering* problem [19]. Filtering techniques deal with the problem of optimally reconstructing a hidden stochastic process at time 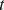 from noisy observations of that process up to time 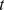. In the situation considered here, the hidden process corresponds to the environment 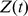, which gets reconstructed from the “observed” history 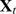 through the conditional mean in Eq. 2.

We assume that the environment 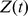 admits a probability distribution 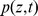 described by a Kolmogorov-forward equation of the form 
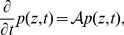
(3)where 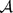 represents the temporal change of 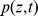, i.e., is the infinitesimal generator of 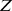. For instance, if 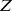 is a diffusion process, 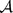 corresponds to the Fokker-Planck operator, while in case of a CTMC, 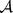 is given by the difference operator of the chemical master equation (CME). In terms of filtering, Eq. 3 corresponds to the process model of 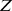. Furthermore, we know that at a given time 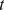, the solution of 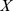 can be written as a sum of independent but time-transformed Poisson processes [20], each of them corresponding to a particular reaction channel. Consequently, the observation model is given by a set of Poisson counting observations with the hazard functions given in Eq.1. This is closely related to Markov-modulated Poisson processes [21] and their corresponding optimal filtering [22].

While a more general treatment is provided in S1 Text, we assume in the following that a one-dimensional process 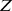 is modulating 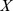 through its 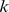-th reaction of order zero. We further restrict ourselves to the case where 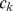 is a linear function of 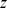, i.e., 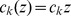. Under those assumptions, it can be shown that the conditional process 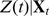 follows a filtering distribution 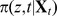 with 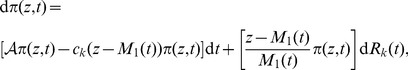
(4)with 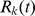 as the number of reactions of type 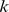 up to time 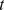 in 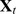 and 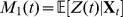 (for a derivation see S.2 in S1 Text). Since Eq. 4 shows an implicit dependency on its own mean, it is complicated to handle numerically. A simpler equation can be obtained for an unnormalized variant 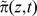 of the filtering distribution [23], i.e.,
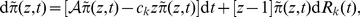
(5)with 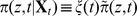 and 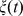 a time-dependent normalizing factor independent of 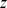. Thus, Eq.5 describes a scaled version of the normalized filtering distribution from Eq.4. However, once 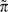 has been numerically solved for, it can be easily rescaled such that it integrates (or sums up) to one for all 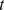.

Note that Eq. 5 is a stochastic partial differential equation (SPDE) in case 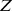 describes a diffusion process or a stochastic difference-differential equation (SDDE) if 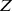 is a CTMC. In the latter case, the solution of Eq.5 can be compactly written as 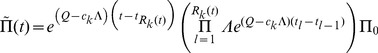
(6)with 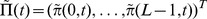, 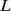 the number of reachable states of 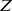, 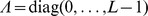, 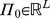 the initial distribution over 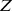 and 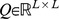 the generator matrix of 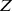. Note that we define 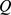 to be a left stochastic matrix, i.e., its rows sum up to zero.

#### Conditional moment dynamics

In order to evaluate Eq. 2, we only require the mean (i.e., the first moment) of the filtering distribution, i.e., 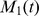. In general, however, the mean also depends on the second-order moment, which in turn depends on the third-order moment and so forth. Generally, the 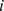-th order non-central moment is found by multiplying both sides of Eq.4 with 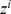 and summing (or integrating) over all 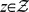, i.e., 
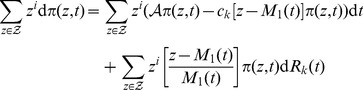
(7)such that the filtering moment dynamics up to order 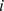 can be generally written as
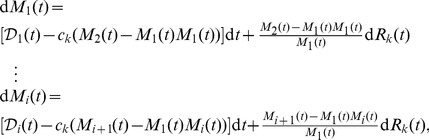
(8)with 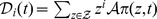 as the unconditional moment dynamics. The computation of moments in case of multivariate environments is performed analogously. Although Eq.8 is generally infinite-dimensional, there are several relevant scenarios, for which the moment dynamics are *closed*, i.e., only depend on higher-order moments up to a certain order. This is for instance the case, if 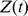 is a Cox-Ingersoll-Ross (CIR) process [24] or any finite state Markov chain. On the other hand, if the moment dynamics are infinite-dimensional, suitable assumptions on the filtering distribution 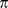 can be imposed to yield a closed moment-dynamics (see [25] and S.3 in S1 Text). An important closure is found by analyzing Eq. 6: especially for large 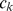 we have that 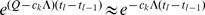 and furthermore,
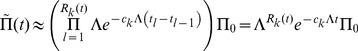
(9)suggesting that Eq. 6 can be well approximated by a Gamma-distribution. We note that the Gamma-distribution is fully characterized by two parameters – or equivalently – its first two moments 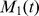 and 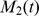. As a consequence, we may express the third order moment as a function of the first two moments, i.e., 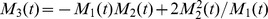, such that the second conditional moment closes as



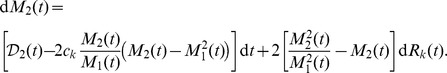



Further discussion on moment-closure is provided in section S.3 in S1 Text. In the following, we demonstrate the uncoupled dynamics using several numerical and analytical case studies.

### Uncoupled dynamics for network simulation

#### The marginal simulation algorithm (MSA)

Although the uncoupled dynamics of 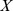 are non-Markovian, the Markov property can be enforced by virtually extending the state space by the first moment of Eq. 8, summarizing the history of 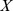. As a result, one can simulate sample paths of the uncoupled process using standard methods that can account for the explicit time-dependency of the hazard functions [26]. In general, such algorithms rely on the generation of random waiting times for each of the reaction channels. All reactions that are independent of 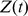 will retain their exponentially distributed waiting times. In contrast, the time 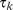 that passes until a reaction of type 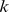 happens is distributed according to 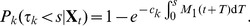
(10)


We note that as long as no reaction of type 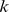 happens, 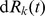 is zero and hence, 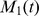 is found by solving a set ordinary differential equations (ODEs). Since that solution is not generally known in closed form, we cannot directly sample from Eq. 10. However, several efficient solutions to that problem have been developed in the context of inhomogeneous Poisson processes, e.g., such as the method of *thinning* [27]. Once a reaction has fired, the filtering moments need to be updated by the terms multiplying the firing process 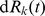 in Eq. 8 (i.e., they exhibit a discontinuity). The following lines describe a possible implementation of the MSA.

**Algorithm 1** (Marginal simulation algorithm) *The algorithm simulates the uncoupled dynamics of a reaction network with* 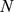 *reactions associated with stoichiometric change vectors* 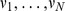 *. The* 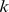 *-th reaction is assumed to be driven by the environmental network* 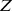 *. The algorithm requires a real-valued constant* 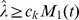 *to be given as an input.*

*1 Initialize variables* 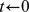 *and* 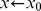.

*2* ***while*** 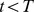 ***do***

*3* ***for*** 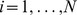 ***do***

*4* ***if*** 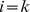 (*marginal hazard*) ***then***

*5   Initialize* 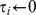 *and* 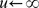.

*6* ***while*** 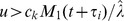 ***do***

*7   Simulate* 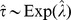.

*8   Set* 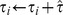.

*9   Simulate* 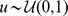.

*10* ***end while***

*11* ***else***

*12  Simulate exponential waiting-time* 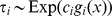.

*13* ***end if***

*14* ***end for***

*15 Choose reaction associated with the minimal waiting-time* 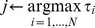.

*16 Update state* 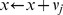.

*17 Update time* 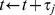.

*18* ***if*** 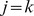 ** ***then***

*19  Update* 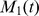 *by the terms accompanying* 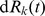 *in Eq. 8*.

*20* ***end if***

*21 Output* 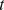 *and* 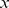.

*22* ***end while***

Evidently, simulation from Eq. 10 (i.e., through the thinning method in lines 5-10 of Algorithm 1) comes at higher cost than simulating from an exponential distribution, since in general, it relies on the numerical integration of an ODE. However, reactions associated with the environmental part no longer need to be simulated, which yields a significant reduction in computational effort as soon as the environmental network is large and expensive to simulate due to high propensity reactions, for instance.

#### An illustrative example

We will now instantiate the proposed framework using a simple telegraph model of transcription, i.e., 
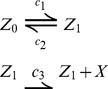
with propensities given by the law of mass action. The two-state promoter 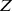 stochastically switches between its inactive state 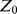 and its active state 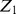, whereas only the latter allows transcription of mRNA (denoted by 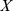). For the sake of illustration, this model is now understood as a process 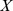 which is driven by an environmental perturbation 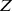. We thus aim to find the uncoupled dynamics of 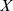, where fluctuations in 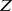 have been marginalized.

In order to evaluate the marginal hazard function, we need to compute the conditional expectation 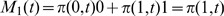. It is straightforward to show that the unconditional probability distribution over the promoter states satisfies the CME 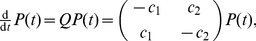
(11)with 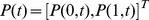. From equation Eq. 5 we further know that the unnormalized conditional probability 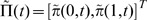 admits the linear stochastic differential equation



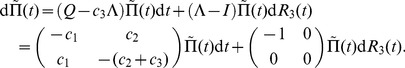
(12)As long as no transcription event takes place (i.e., 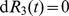), the conditional distribution from Eq.12 is just given by a linear homogeneous ODE, whose solution is known in closed form. We refer to this distribution as 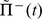. In case a reaction happens at time 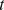, the distribution will be updated according to 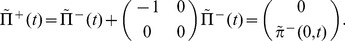
(13)


**Figure 2:**
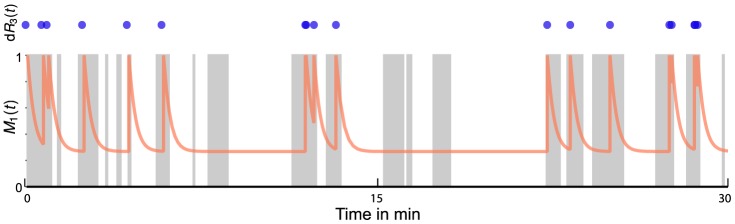
Schematic illustration of the marginal simulation algorithm. The red line shows the computation of the marginal hazard function 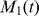 of the telegraph model (i.e., using Eq.14). The blue dots indicate the corresponding transcription events. Also shown is a possible corresponding realization of the promoter state (gray bars indicate the time the promoter has been active). The figure illustrates that using the MSA algorithm, only the events associated with X need to be simulated (in this case the transcription of mRNA). In contrast, SSA requires explicit simulation of all environmental states, which – depending on the time-scale – may become computationally prohibitive.

Upon normalization of the distribution 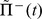 we may express its expectation between two consecutive firing times 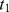 and 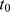 with 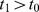 as 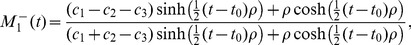
(14)with 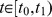 and 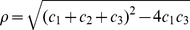. Furthermore, normalization of 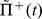 shows that when the next reaction at time 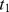 fires, the conditional expectation instantaneously changes to one. This is consistent with the fact that for a transcription event to happen at 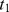, the promoter must be in its active state at least until 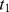. Fig. 2 illustrates the computation of the marginal hazard function used during MSA.

Another important quantity to describe the uncoupled model is its Kolmogorov-forward- (or master-) equation. We remark that since the uncoupled dynamics are non-Markovian, they do not satisfy a conventional master equation. Instead, such processes are described by *generalized master equations* (GME) that can account for memory effects in the dynamics (see S.5 in S1 Text for further discussion). In particular, there exists a fundamental relation between a GME's memory and the corresponding network's waiting-time distributions [28]. For the uncoupled telegraph model, the waiting-time distribution 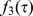 of the transcription events is fully tractable using Eq. 10 (for an explicit expression see S1 Mathematica Notebook). It is therefore straightforward to develop a Chapman-Kolmogorov type of equation for the transcriptional dynamics 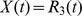, i.e., 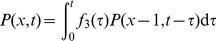
(15)for 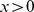 and 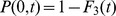 with 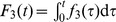 as the cumulative waiting-time distribution. Following [28], Eq. 15 can be transformed into a gain-loss-type of master equation, i.e.,
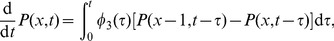
(16)where the memory kernel 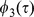 is related to 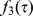 through the *Montroll-Weiss* equation (see S.5 in S1 Text). Eq. 16 represents the desired marginal master equation, from which further analysis could be deduced.

#### Comparison of MSA to existing approaches

**Figure 3:**
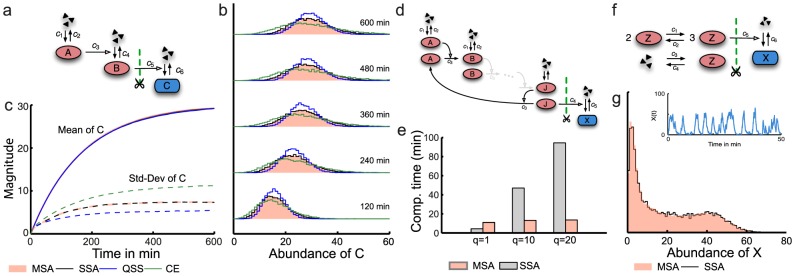
Evaluation of the marginal simulation algorithm. (a) Simple three-stage model. Species A, B and C are modeled as coupled linear birth-death processes. Filled arrows correspond to mass-action reactions, whereas empty arrows connecting species S and reaction 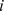 indicate that S linearly modulates the rate of that reaction, i.e., 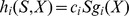. The rate constants were chosen to be 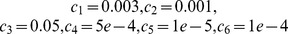. The uncoupled marginal dynamics of C are obtained under a second-order zero-cumulants closure. (b, c) Evaluation of the marginal simulation algorithm. Simulations based on the QSS-approximation neglect a significant portion of variability as opposed to assuming a constant environment (CE) in which case the variability is overestimated. In contrast, the uncoupled dynamics correctly predict the fluctuations on the protein level, while yielding a reduction in computational effort when compared to standard SSA (i.e., a simulation time of approximately 20 min instead of 46 min). For each of the schemes, 7000 sample paths have been used to compute the respective histograms (and moments). (d) Nonlinear reaction network. A ten-dimensional reaction network consisting of species (A, B,…, J) modulates the production rate of a birth-death process X (rate constants 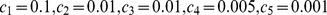). (e) The uncoupled dynamics of X were computed under a second-order zero-cumulants closure. Different environmental time-scales were realized by multiplying the vector of rate constants by a constant factor 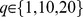. For each 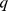, 2000 samples paths were simulated to assess the performance of MSA and SSA, respectively. In all cases, the estimated Kolmogorov distance between the SSA and MSA distributions was below 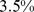. (f) Bistable reaction network. The environmental part was modeled as a one-dimensional Schloegl system [33] modulating the production rate of a birth-death process X (rate constants 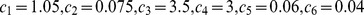). (g) The uncoupled dynamics were computed by integrating Eq.5 on a truncated state space (i.e., feasible states 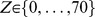). The distribution over X was computed at 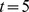 minutes using 50000 MSA and SSA samples (estimated Kolmogorov distance of 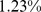). The computation of 50 MSA and SSA samples took 13.79 and 255.05 seconds, respectively.

The impact of environmental fluctuations on a dynamical system of interest is as diverse as the timescale on which they operate. For instance, extrinsic noise in the context of gene expression might be slowly varying (e.g., correlates well with the cell-cycle [29], [30]), while fluctuations in transcription factor abundance might be significantly faster than the expression kinetics downstream. From a technical point of view, timescales range from constant environmental conditions that are random but fixed [31] to regimes where the fluctuations are very fast, such that quasi-steady-state (QSS) assumptions become applicable [16], [32]. A QSS-based approach for simulating a system 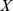 in the presence of extrinsic noise 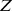 corresponds to simulating the conditional CTMC 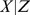, where 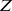 is replaced by the mean of 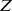. Alternatively, one may try to replace a fluctuating environment 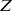 through a random but fixed environment of same variance but this leads to an overestimation of the process variance in 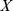 [5], as discussed in a later section. To investigate the two above simplifying assumptions and compare them to the exact solution obtained via SSA and MSA, we performed a simulation study on a linear three-stage birth-death model given in Fig. 3a. In this case only species *C* is considered of interest whose uncoupled dynamics are obtained by marginalizing *A* and *B*. The results from Fig. 3b and Fig. 3c show that MSA facilitates accurate and fast approximations also under intermediate environmental time-scales where QSS- and static environmental assumptions break down.

#### Varying environmental time-scales

Simulation of the joint system 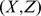 becomes particularly challenging if the environmental fluctuations are fast, while with Eq. 8 the complexity of the marginal process simulation is invariant with respect to the time-scale of the environment. To demonstrate this effect, we performed a simulation study using a ten-dimensional, non-linear environmental network (Fig. 3d). Different time-scales were realized by multiplying the vector of environmental rate constants by a constant factor 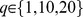, effectively changing the number of reactions that have to be simulated on average. The results from Fig. 3e demonstrate that the computation time of the SSA simulation strongly increases with 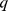. In contrast, the MSA's efficiency appears largely invariant with respect to the environmental time-scale, possibly leading to a substantial reduction in computational effort. We remark that for all three time-scales, the MSA algorithm achieved very high accuracies (i.e., estimated Kolmogorov distances below 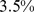). However, If the environmental time-scale is comparably slow (i.e., if 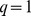), the extra effort needed for computing the marginal hazard function dominates, in which case SSA appears favorable.

#### Bistable environmental fluctuations

We further analyzed the case where the environmental network exhibits a more complex dynamics. In particular, we considered a bistable Schloegl system 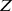 [33] modulating the production rate of a birth-death process 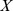 (Fig. 3f). Here the most efficient way to compute the marginal hazard functions was to directly integrate Eq. 5 under a suitable state space truncation (see caption of Fig. 3g for fuller details). The results shown in Fig. 3g indicate that also in this case, the MSA algorithm achieves high accuracies but at the same time finishes around 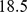 times faster than the corresponding SSA algorithm. These results underpin the significance of MSA in cases where the environmental network is costly to simulate (i.e., many reactions take place) but at the same time, a QSS-based approach is unable to capture the correct dynamics (e.g., a bistable production rate).

### Uncoupled dynamics for network analysis

#### Propagation of environmental fluctuations and the effective noise

Several recent studies [34], [5], [6], [4] are centered around the separation of different noise contributions in biochemical networks. Typically, the law of total variance is employed to decompose the fluctuations of 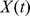 into parts that are intrinsic to 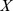 and parts that come from 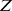 (i.e., are extrinsic to 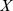). Here we found that performing such an analysis on 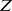 instead of 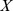 – in conjunction with our decoupling approach – provides a novel way to study how stochasticity is propagated through biochemical networks. Using the law of total variance, we can decompose the total (or unconditional) variance of 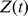 as 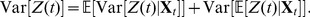
(17)


The two terms on the r.h.s. can be interpreted as follows. Assume we can observe 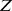 only through 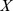. Since 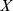 is intrinsically stochastic, a part of the variability of 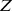 is not carried over to 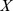. In Eq. 17, this part (i.e., the *suppressed noise*) corresponds to the first term on the r.h.s. since it quantifies the uncertainty about 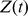 that remains after observing 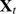. The second term determines how accurate 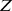 can be reconstructed from trajectories 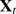. Alternatively, it can be understood as the amount of noise in 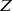 that effectively impacts 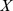 (i.e., the *effective noise*). For instance, the environmental process could be characterized by a large variance, but still have only marginal impact on 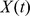 – depending on the timescale of 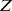 and 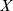.

In order to quantify those terms, we note that the conditional variance within the first term coincides with the second-order central moment of the filtering distribution from Eq.5. This further implies that it can be computed “on-the-fly” when simulating 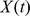 using the marginal simulation algorithm which allows an efficient estimation of its expectation. However, in some biologically relevant cases, the effective noise can be determined even analytically, which we demonstrate in the following.

We derive in section S.4 in S1 Text that the expected central moments are generally given by 
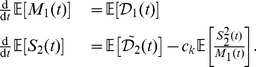
(18)


**Figure 4:**
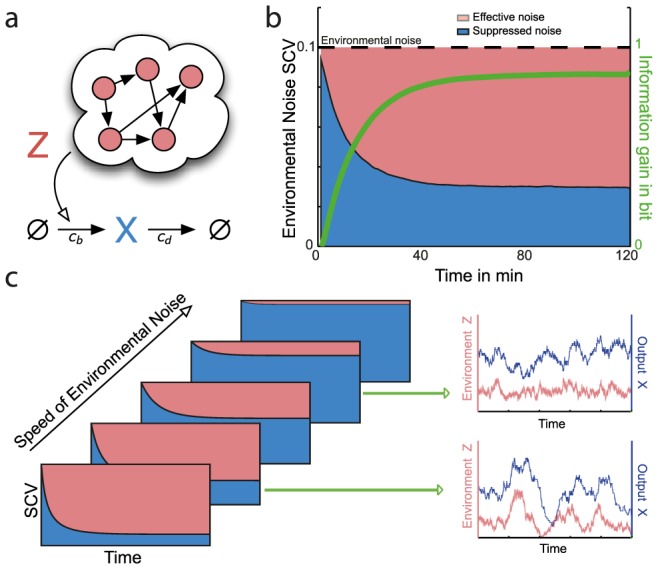
Propagation and suppression of environmental fluctuations. (a) Linear birth-death process in a fluctuation environment. The birth-rate is assumed to be linearly modulated by its environment 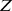 modeled as a CIR process (see Eq. 19). (b) Calculation of suppressed and effective noise. Individual components were computed analytically by solving an ordinary differential equation (see Eq. 22 in S1 Text) with 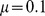, 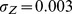, 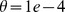 and 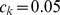. For orientation, we also show the information gain between 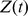 and 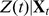, computed using the marginal simulation algorithm (green); it can be understood as the gain in information about 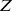 through observing 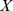 and it exhibits a monotone relationship with the effective noise. (c) Relation between the effective noise and the speed of the environmental fluctuations. Noise contributions were computed by numerically solving Eq. 22 in S1 Text for 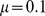, 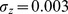, 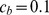 and different values of 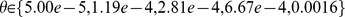. Note that the results are independent of the death rate 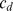. Also shown are realizations of 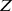 and 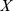 for the case for two different time-scales. If 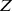 is fast, the output 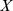 is able to suppress most of the variability in 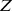. In contrast, if 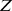 is slow, fluctuations are largely transferred to 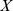 (i.e.,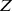 has a large effective noise) such that extrinsic noise in 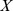 is substantial.

The mean in Eq.18 is just the unconditional mean of 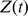, while the derivative of the expected variance shows an additional negative term, causing it to be smaller than the unconditional variance. Let us for instance consider the case where 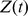 follows a Cox-Ingersoll-Ross process governed by the SDE 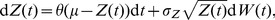
(19)with 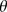, 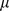 and 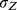 as real process parameters and 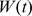 as a standard Wiener process. Note that in this case, Eq. 18 reduces to an autonomous ODE, which for large 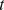 yields the relative effective noise 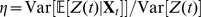 at stationarity, i.e.,
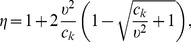
(20)where 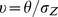 can be considered a normalized timescale of 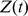 (see section S.4 in S1 Text). The computation of the effective noise and its dependency on the environmental timescale is illustrated in Fig. 4.

#### The slow noise approximation (SNA)

The effective noise can be understood as a measure of how strong 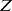 impacts 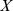. Only in the special case of a very slow or constant environment, i.e., 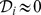, it turns out that for large 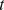, 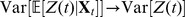, i.e., all variability in 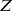 is transferred to 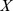. Hence, a more noisy but fluctuating environment may induce a similar (or even the same) effective noise in 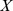 as a random but fixed environment of the same variance. Consequently, when looking at only snapshot data for 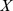 one can generally not infer whether the environment is constant or fluctuating. On the other hand, this implies that we may well approximate the impact of a complicated and dynamically changing environment by a simple random variable of appropriate variance. More specifically, we demand for an equivalent constant environment 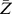 such that 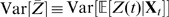, where 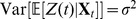 is the effective noise of the original, fluctuating environment 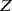 at stationarity. Let us again consider the birth-death process of Fig. 4a and set the birth rate to one such that any scaling is subsumed in the environmental process 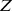. With 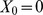, the abundance of the birth death process at any time is given by 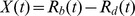 with 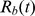 and 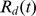 as counting processes for the birth and death reaction, respectively. We show in section S.5.1 in S1 Text that the marginal birth hazard is approximately given by 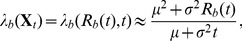
(21)with 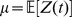 the unconditional mean and 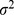 the effective noise of 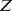, whereas the expression becomes exact for constant and infinitely fast environments. Note that the marginal hazard does not depend on the full history, but only the number of birth-reactions 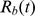 up to time 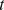. That is, 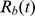 is a sufficient statistic for evaluating the conditional expectation 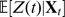. In relation to QSS, which assumes that no fluctuations of 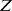 are propagated to 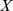, the found equivalent constant environment with the proper effective noise provides a better approximation for a decoupled simulation of environment and process of interest than QSS.

**Figure 5:**
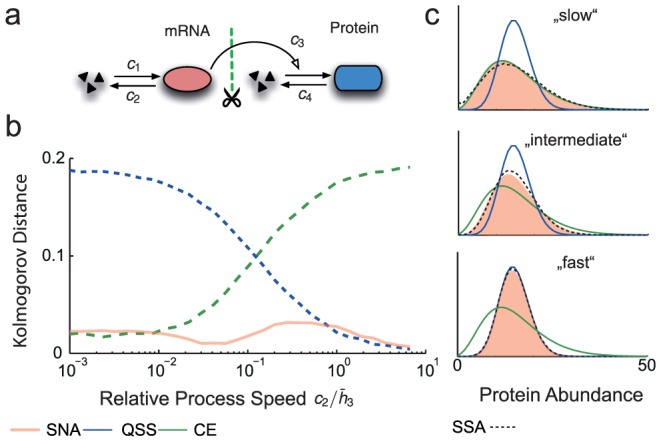
Analytical protein distributions through the slow noise approximation. (a) Two-stage gene expression model. Transcription and translation are modeled through mass-action kinetics with reaction rate constants 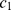-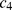. Fluctuations on the mRNA are considered environmental and hence, integrated out in order to obtain a one-dimensional stochastic process describing only the protein. (b) Accuracy of the slow noise approximation. The SNA was compared to the QSS- and CE-approximations by means of the Kolmogorov distance between the respective approximate and exact distribution (SSA) as a function of the relative speed of the mRNA fluctuations defined by 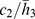 and 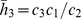. The mRNA birth- and death-rates were varied between 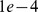 and 1 while maintaining a constant ratio of 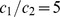. The remaining parameters were chosen as 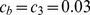 and 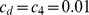. QSS- and CE approximations break down for slow or fast environmental fluctuations respectively, whereas the SNA yields accurate distributions regardless of the mRNA's timescale. (c) Exemplary distributions obtained through the different approaches in three different regimes (slow, intermediate, fast).

Using the effective noise, we now aim to find a master equation, which describes the time-evolution of the marginal probability distribution 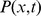. Since 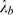 depends on 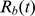 rather than 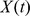, it appears natural to formulate the master equation in 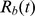 and 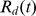 as well. For the example considered here, one can show that the probability distribution 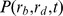 satisfies a GME of the form 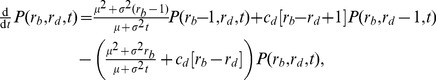
(22)that can be solved analytically using generating functions (see S.5.1 in S1 Text). From 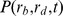 we compute the distribution of 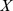 as
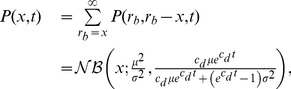
(23)i.e., a negative binomial distribution. Eq. 23 provides a surprisingly simple approximate solution for the transient probability distribution of birth death processes in a fluctuating environment. In order to check its validity, we compared the analytical approximate distributions to the ones obtained through SSA for a gene expression model, where the environmental fluctuations are assumed to be due to the mRNA dynamics (see Fig. 5). More specifically, we computed the Kolmogorov distance between the resulting protein distributions as a function of the environmental timescale. Apart from the exact correspondence for the limiting time-scales, Fig. 5 indicates that the SNA provides a good approximation regardless of the environmental timescale.

## Discussion

There is increasing evidence that models of biochemical networks need to account for both intrinsic and extrinsic noise caused by variations in the intracellular environment. In recent studies, this is done by extending a model's state space by certain environmental species, whose dynamics are described along with the actual system of interest. In particular, the resulting system dynamics are described and studied *conditional* on a particular history of the environment and thus, do not provide a coherent description of a dynamical system subject to extrinsic noise. In this work, we derived and analyzed a novel process framework, which is able to describe just the system of interest as if it was still embedded into its environment. In that sense, it permits a mathematically exact way to analyze small parts of networks in an *uncoupled* fashion.

Several recent studies rely on the extreme assumptions that the environmental fluctuations are either infinitely fast or slow. While both strategies may in fact lead to strongly simplified and tractable models, they are characterized by significant approximation errors when considering intermediate environmental timescales (see e.g., Fig. 5b). The approach proposed here allows to uncouple a reaction network from its surrounding environment regardless of the latter's timescale. In that sense, the approach is fully general although practical implementations may rely on efficient but approximate solutions of the discussed filtering problem.

In the context of Monte Carlo simulation the decoupled process can yield a significant reduction in computational effort when compared to standard SSA – especially if the environmental network is costly to simulate. This highlights the role of the provided framework as a general tool to split stochastic biochemical networks into individual parts that are easier to simulate. We believe that it will aid in turning stochastic modeling and simulation techniques more large-scale and more faithful to *in vivo* conditions, where significant environmental fluctuations are present. Moreover, the framework can be used in the model-based design of novel circuit motifs in synthetic biology and is related to the notion of retroactivity [35].

We further demonstrate that the uncoupled dynamics provide a novel analytical tool to study how environmental stochasticity is propagated along coupled reaction networks. For instance, we have shown that the total environmental noise splits up into two terms: one corresponding to the noise that is suppressed and a second term that quantifies the effective noise that is *sensed* by the target network. In [36] the authors derive a lower bound on a network's ability to suppress fluctuations and show its immediate relation to the uncertainty at which those fluctuations can be estimated – similar to what we defined as effective noise. The methods proposed here allow to not only bound, but fully determine both the suppressed and effective environmental noise. Our results further indicate that two environments with very different timescales may impact a network in a similar way. For instance a fixed but random environment may yield the same effective noise as a fluctuating environment with larger variance. Along those lines, we derived a simple but widely applicable approximation of the transient probability distribution for birth-death processes subject to environmental noise. It is based on the idea to approximate a fluctuating environmental process by a simple random variable that impacts the birth death process in an equivalent way. In order to solve for the transient probability distribution we derived a generalized master equation for this non-Markovian process. The results were derived under the assumption of a one-dimensional environmental process but its extension to multivariate scenarios shall be examined at a later stage.

The presented framework provides a novel and orthogonal notion of environmentally perturbed stochastic networks from which several interesting avenues could be pursued. However, the approach in its current form has a few practical limitations that should be addressed in future work. Above all, if the uncoupled dynamics cannot be computed analytically, its accuracy – and hence, reliability will depend on finding suitable approximate schemes. For that sake, we believe that one will profit from the approach's tight connection to stochastic filtering – a field that has been thoroughly explored over the past decades. Therefore, the marginal hazard functions may be approximated by exploiting certain features of the associated filtering problems such as their asymptotic theories and so forth. Those could for instance be used to derive more general moment-closure schemes or to construct concise and more efficient state-space truncations [37] when integrating the respective filtering distribution. The main bottleneck of the MSA algorithm is the simulation of the non-exponential waiting-times, which relies on the integration of an ODE. Therefore, the use of optimized and dedicated ODE solvers could further boost the algorithm's performance.

## Models

All models and simulations were implemented in MATLAB (The MathWorks, Natick, MA). Source codes for reproducing the numerical results have been attached as S1 Database.

## Supporting Information

S1 Text Supplementary theory and derivations.
(PDF)

S1 Mathematica Notebook Mathematica notebook for the telegraph model.
(NB) Click here for additional data file.

S1 Database MATLAB source codes used for simulations.
(ZIP)
