# Supplementary material for: Uncoupled Analysis of Stochastic Reaction Networks in Fluctuating Environments
Source: PLoS Comput Biol. 2014 Dec 4;10(12):e1003942. doi: 10.1371/journal.pcbi.1003942 (PMC4256010; doi:10.1371/journal.pcbi.1003942)
Supplement: S1 Text — Supplementary theory and derivations. (PDF) [file pcbi.1003942.s001.pdf]

Uncoupled analysis of stochastic reaction networks in fluctuating environments

Supporting Text S1

C. Zechner and H. Koeppl

S.1 Derivation of the uncoupled dynamics

The hazard function of the uncoupled reaction network corresponding to the environmental process Z is obtained through the innovation theorem for counting processes [1, 2] and ref. [17] in the main text. For static environments, such reaction hazards have been derived in (ref. [9] in the main text and [9]) but an equivalent calculation is possible in the case of fluctuating environments. For completeness, we provide a simple derivation in the following. Conceptually, the construction of such a process can be understood as a *marginalization* of the process dynamics with respect to Z. The critical point is to anticipate that the marginal process will depend on the full process history $\mathbf{X}_t$. We can then write the marginal jump probability of the k-th reaction as

$$\begin{aligned}
P(X(t+\Delta t) = x + \nu_k \mid \mathbf{X}_t) &= \int P(X(t+\Delta t) = x + \nu_k \mid Z(t) = z, X(t) = x) p(Z(t) = z \mid \mathbf{X}_t) \mathrm{d}z \\
&= \int \Delta t c_k(z) g_k(x) p(Z(t) = z \mid \mathbf{X}_t) \mathrm{d}z \\
&= \Delta t \mathbb{E}\left[c_k(Z(t)) \mid \mathbf{X}_t\right] g_k(x) \\
&= \lambda_k(\mathbf{X}_t)\Delta t.
\end{aligned} \tag{1}$$

S.2 Derivation of the normalized filtering distribution

Given the posterior distribution over Z at time t, the one-step posterior for a sufficiently small Δt can be generally written as (ref. [22] in the main text)

$$p(Z(t+\Delta t) = z \mid \Delta R_k = \{0,1\}, \mathbf{X}_t) = \frac{p(\Delta R_k = \{0,1\} \mid Z(t+\Delta t) = z) p(Z(t+\Delta t) = z \mid \mathbf{X}_t)}{\sum_{z=0}^{\infty} p(\Delta R_k = \{0,1\} \mid Z(t+\Delta t) = z) p(Z(t+\Delta t) = z \mid \mathbf{X}_t)}, \tag{2}$$

where ΔR_k indicates whether a reaction has happened in Δt or not. The likelihood term in (2) is given by the Poissonian observation model with propensity $h_k(X(t), z) = c_k z g_k(X(t))$, i.e.,

$$p(\Delta R_k = 0 \mid Z(t+\Delta t) = z) = e^{-h_k(X(t),z)\Delta t} \tag{3}$$

$$p(\Delta R_k = 1 \mid Z(t+\Delta t) = z) = \Delta t h_k(X(t), z) e^{-h_k(X(t),z)\Delta t}. \tag{4}$$

For small Δt, we can write the one-step prior distribution as

$$p(Z(t+\Delta t) = z \mid \mathbf{X}_t) \approx p(Z(t) = z \mid \mathbf{X}_t) + \Delta t \mathcal{A} p(Z(t) = z \mid \mathbf{X}_t). \tag{5}$$

Hence, in case no reaction happens, we obtain

$$\begin{aligned}
p(Z(t+\Delta t) = z \mid \Delta R_k = 0, \mathbf{X}_t) &= \frac{e^{-c_k z g_k(X(t))\Delta t} \left[p(Z(t) = z \mid \mathbf{X}_t) + \Delta t \mathcal{A} p(Z(t) = z \mid \mathbf{X}_t)\right]}{\sum_{z=0}^{\infty} e^{-c_k z g_k(X(t))\Delta t} p(Z(t+\Delta t) = z \mid \mathbf{X}_t)} \\
= \frac{e^{-c_k z g_k(X(t))\Delta t} p(Z(t) = z \mid \mathbf{X}_t)}{\sum_{z=0}^{\infty} e^{-c_k z g_k(X(t))\Delta t} p(Z(t+\Delta t) = z \mid \mathbf{X}_t)} &+ \frac{\Delta t \mathcal{A} p(Z(t) = z \mid \mathbf{X}_t)}{\sum_{z=0}^{\infty} e^{-c_k z g_k(X(t))\Delta t} p(Z(t+\Delta t) = z \mid \mathbf{X}_t)}
\end{aligned} \tag{6}$$

Taking the limit yields the temporal change in p, i.e.,

$$\begin{aligned}
&\lim_{\Delta t \to 0} \frac{p(Z(t+\Delta t) = z \mid \Delta R_k = 0, \mathbf{X}_t) - p(Z(t) = z \mid \mathbf{X}_t)}{\Delta t} \\
&\quad = \lim_{\Delta t \to 0} \frac{p(Z(t) = z \mid \mathbf{X}_t) \left[e^{-c_k z g_k(X(t))\Delta t} - \sum_{z=0}^{\infty} e^{-c_k z g_k(X(t))\Delta t} p(Z(t+\Delta t) = z \mid \mathbf{X}_t) \right]}{\Delta t \sum_{z=0}^{\infty} e^{-c_k z g_k(X(t))\Delta t} p(Z(t+\Delta t) = z \mid \mathbf{X}_t)} \\
&\quad + \lim_{\Delta t \to 0} \frac{\mathcal{A} p(Z(t) = z \mid \mathbf{X}_t)}{\sum_{z=0}^{\infty} e^{-c_k z g_k(X(t))\Delta t} p(Z(t+\Delta t) = z \mid \mathbf{X}_t)} \\
&\quad = p(Z(t) = z \mid \mathbf{X}_t) \left[-c_k z g_k(X(t)) + c_k g_k(X(t)) \mathbb{E}\left[Z(t) \mid \mathbf{X}_t \right] \right] + \mathcal{A} p(Z(t) = z \mid \mathbf{X}_t) \\
&\quad = \mathcal{A} p(Z(t) = z \mid \mathbf{X}_t) + c_k g_k(X(t)) \left[z - \mathbb{E}\left[Z(t) \mid \mathbf{X}_t \right] \right] p(Z(t) = z \mid \mathbf{X}_t).
\end{aligned} \tag{7}$$

Using a simpler notation, we can write the differential change in case no reaction happens as

$$\dot{p} = \mathcal{A} p - c_k g_k(X(t)) \left[z - M_1 \right] p, \tag{8}$$

with M_1 as the posterior expectation of Z. If one reaction happens, we can write the one-step posterior distribution as

$$\begin{aligned}
p(Z(t+\Delta t) = z \mid \Delta R_k = 1, \mathbf{X}_t) = &\frac{c_k z g_k(X(t)) e^{-c_k z g_k(X(t))\Delta t} p(Z(t) = z \mid \mathbf{X}_t)}{\sum_{z=0}^{\infty} c_k z g_k(X(t)) e^{-c_k z g_k(X(t))\Delta t} p(Z(t+\Delta t) = z \mid \mathbf{X}_t)} \\
&+ \frac{\Delta t \mathcal{A}(p(Z(t) = z \mid \mathbf{X}_t))}{\sum_{z=0}^{\infty} c_k z g_k(X(t)) e^{-c_k z g_k(X(t))\Delta t} p(Z(t+\Delta t) = z \mid \mathbf{X}_t)}.
\end{aligned} \tag{9}$$

Since the posterior jumps instantaneously when the reaction happens, the derivatives are not defined. Instead, we compute the increments if Δt approaches zero. This yields

$$\begin{aligned}
&\lim_{\Delta t \to 0} p(Z(t+\Delta t) = z \mid \Delta R_k = 1, \mathbf{X}_t) - p(Z(t) = z \mid \mathbf{X}_t) \\
&\quad = \lim_{\Delta t \to 0} \frac{c_k z g_k(X(t)) e^{-c_k z g_k(X(t))\Delta t} p(Z(t) = z \mid \mathbf{X}_t)}{\sum_{z=0}^{\infty} c_k z g_k(X(t)) e^{-c_k z g_k(X(t))\Delta t} p(Z(t+\Delta t) = z \mid \mathbf{X}_t)} - p(Z(t) = z \mid \mathbf{X}_t) \\
&\quad = \lim_{\Delta t \to 0} \frac{z e^{-c_k z g_k(X(t))\Delta t} p(Z(t) = z \mid \mathbf{X}_t)}{\sum_{z=0}^{\infty} z e^{-c_k z g_k(X(t))\Delta t} p(Z(t+\Delta t) = z \mid \mathbf{X}_t)} - p(Z(t) = z \mid \mathbf{X}_t) \\
&\quad = \left[\frac{z - M_1}{M_1} \right] p
\end{aligned} \tag{10}$$

Finally, the overall time-evolution of p is given by the stochastic equation

$$\mathrm{d}p = \Big(\mathcal{A} p - c_k g_k(X(t)) \left[z - M_1 \right] p \Big) \mathrm{d}t + \left[\frac{z - M_1}{M_1} \right] p \mathrm{d}R_k. \tag{11}$$

Note that this equation depends on the conditional mean of Z, such that it becomes tedious to solve directly. In contrast, the unnormalized density provided above can be solved and normalized in a subsequent step. Nevertheless, the normalized distribution allows a simple computation of the posterior moments of Z.

S.3 Approximate moment dynamics

In cases where the conditional process $Z(t) \mid \mathbf{X}_t$ cannot be fully characterized by a finite number of moments, one can employ suitable approximations to obtain a closed moment system (e.g., ref. [24] in the main text). Such schemes – commonly referred to as *moment-closure* – aim to approximate the moment dynamics of a system using certain distributional assumptions. It has been shown that moment-closure techniques may yield reasonable approximation performances in several practical scenarios (see e.g., ref. [8] in the main text). In other cases – however – they might not be able to correctly capture the desired moments and moreover, there is no principled way of assessing the quality of a particular closure

"beforehand", i.e., without performing extensive stochastic simulations. Those problems are inherent if one aims to approximate $Z(t)$ without including further information. In contrast, the conditional process $Z(t) \mid \mathbf{X}_t$ turns out to be more straight-forward to approximate. This stems from the fact that due to the conditioning, the distribution over $Z(t)$ will generally be more informative than the unconditional distribution. This can be understood via Bayes' theorem: a complicated and broad prior distribution is significantly harder to approximate by a simple distribution (e.g., a Gaussian) than a posterior distribution that is obtained after observing data. The more data (i.e., information) is included, the tighter and symmetric it is. While such arguments appear largely qualitative, they can be rigorously formulated using concepts from asymptotic theory such as large sample properties of Bayesian estimators [4]. In fact, our simulations indicated that the approximation accuracy of the uncoupled dynamics often shows little sensitivity with respect to the particular closure function. For univariate environments, we consistently used the Gamma-type of closure described in the main text. For the multivariate case, we applied the second-order *zero-cumulant* closure in which the third order moments are approximated by the first- and second-order moments as

$$\mathbb{E}\left[ABC\right] = \mathbb{E}\left[A\right]\mathbb{E}\left[BC\right] + \mathbb{E}\left[B\right]\mathbb{E}\left[AC\right] + \mathbb{E}\left[C\right]\mathbb{E}\left[AB\right] - 2\mathbb{E}\left[A\right]\mathbb{E}\left[B\right]\mathbb{E}\left[C\right]. \tag{12}$$

S.4 Derivation of the effective noise

We assume now that $Z(t)$ modulates $X(t)$ through a zero-order reaction with index k. We have demonstrated in the main text that at any time t the total variance of $Z(t)$ splits up into two terms: (i) the suppressed noise and (ii) the effective noise. In order to quantify the former, we rewrite the conditional moments in terms of *central* instead of non-central moments. In particular, we obtain for the mean and variance

$$\begin{aligned} \mathrm{d}M_1(t) &= \Big(\mathcal{D}_1(t) - c_k S_2(t)\Big)\mathrm{d}t + \frac{S_2(t)}{M_1(t)}\mathrm{d}R_k(t) \\ \mathrm{d}S_2(t) &= \Big(\tilde{\mathcal{D}}_2(t) - c_k S_3(t)\Big)\,\mathrm{d}t + \left[\frac{S_3(t)}{M_1(t)} - \frac{S_2^2(t)}{M_1^2(t)}\right]\mathrm{d}R_k(t) \end{aligned} \tag{13}$$

with $S_2(t)$ as the conditional variance $\mathrm{Var}\left[Z(t) \mid \mathbf{X}_t\right]$ and $\tilde{\mathcal{D}}_2(t)$ as the unconditional central moment dynamics of order two. We next need to compute the expected value of $S_2(t)$. Decomposing $\mathrm{d}R_k(t)$ into a predictable part and a martingale, i.e., $\mathrm{d}R_k(t) = c_k M_1(t)\mathrm{d}t + \mathrm{d}Q_k(t)$, we can rewrite Eq.13 as

$$\begin{aligned} \mathrm{d}M_1(t) &= \mathcal{D}_1(t)\mathrm{d}t + \frac{S_2(t)}{M_1(t)}\mathrm{d}Q_k(t) \\ \mathrm{d}S_2(t) &= \left(\tilde{\mathcal{D}}_2(t) - c_k \frac{S_2^2(t)}{M_1(t)}\right)\mathrm{d}t + \left[\frac{S_3(t)}{M_1(t)} - \frac{S_2^2(t)}{M_1^2(t)}\right]\mathrm{d}Q_k(t) \end{aligned} \tag{14}$$

Taking the expectation of Eq.14, all terms involving $\mathrm{d}Q_k(t)$ become zero and we obtain

$$\begin{aligned} \frac{\mathrm{d}}{\mathrm{d}t}\mathbb{E}\left[M_1(t)\right] &= \mathbb{E}\left[\mathcal{D}_1(t)\right] \\ \frac{\mathrm{d}}{\mathrm{d}t}\mathbb{E}\left[S_2(t)\right] &= \mathbb{E}\left[\tilde{\mathcal{D}}_2(t)\right] - c_k\mathbb{E}\left[\frac{S_2^2(t)}{M_1(t)}\right]. \end{aligned} \tag{15}$$

Although eq. (15) is fully general, it might be hard to evaluate the expectation $\mathbb{E}\left[S_2^2(t)/M_1(t)\right]$ (and possible other further terms stemming from prior dynamics $\tilde{\mathcal{D}}_2(t)$).

S.4.1 Effective noise of a Cox-Ingersoll-Ross process

Let us consider the case where $Z(t)$ follows a CIR process as defined in the main text. The expected central moments are then governed by

$$\begin{aligned}
\frac{\mathrm{d}}{\mathrm{d}t}\mathbb{E}\left[M_1(t)\right] &= \theta(\mu - \mathbb{E}\left[M_1(t)\right])\mathrm{d}t \\
\frac{\mathrm{d}}{\mathrm{d}t}\mathbb{E}\left[S_2(t)\right] &= -2\theta\mathbb{E}\left[S_2(t)\right] + \sigma_Z^2\mathbb{E}\left[M_1(t)\right] - c_k\mathbb{E}\left[\frac{S_2^2(t)}{M_1(t)}\right].
\end{aligned} \tag{16}$$

The only term that remains to be specified is the expectation $\mathbb{E}\left[S_2^2(t)/M_1(t)\right]$. Fortunately, it turns out that for a Gamma-type conditional distribution, this expectation simplifies to $\mathbb{E}\left[S_2^2(t)/M_1(t)\right] = \mathbb{E}\left[S_2(t)\right]^2/\mathbb{E}\left[M_1(t)\right]$. A derivation of that fact can be performed using the extension of Ito's lemma for counting processes. However, since it involves a multitude of technicalities that are not in the scope of this study, we skip the individual steps. Instead we provide a heuristic but substantially simpler explanation based on the fact that the CIR process is *conjugate* to the Poissonian reaction channel. In particular, we consider the case of a Gamma distributed random variable $Z \sim \mathcal{G}(\alpha, \beta)$, with α and β as shape- and inverse scale parameters. The random variable is observed through a Poissonian measurement $X \mid Z = z \sim \mathrm{Poiss}(z)$. After observing X, the conditional distribution over Z is given by

$$p(z \mid X = x) = \mathcal{G}(z; \alpha + x, \beta + 1). \tag{17}$$

Furthermore, the conditional mean and variance are

$$\begin{aligned}
M_1 &= \frac{\alpha + x}{\beta + 1} \\
S_2 &= \frac{\alpha + x}{(\beta + 1)^2}
\end{aligned} \tag{18}$$

and the ratio thereof becomes

$$\frac{S_2^2}{M_1} = \frac{\alpha + x}{(\beta + 1)^3}. \tag{19}$$

Taking the expectation with respect to x then yields

$$\frac{S_2^2}{M_1} = \frac{\alpha + \mathbb{E}\left[X\right]}{(\beta + 1)^3}. \tag{20}$$

We now compare this expression to $\mathbb{E}\left[S_2\right]^2/\mathbb{E}\left[M_1\right]$. In particular, we obtain for the two expectations

$$\begin{aligned}
\mathbb{E}\left[M_1\right] &= \frac{\alpha + \mathbb{E}\left[X\right]}{\beta + 1} \\
\mathbb{E}\left[S_2\right] &= \frac{\alpha + \mathbb{E}\left[X\right]}{(\beta + 1)^2},
\end{aligned} \tag{21}$$

and therefore, the both expressions will coincide. The expected moments – and hence the suppressed noise then can be found by solving

$$\begin{aligned}
\frac{\mathrm{d}}{\mathrm{d}t}\mathbb{E}\left[M_1(t)\right] &= \theta(\mu - \mathbb{E}\left[M_1(t)\right])\mathrm{d}t \\
\frac{\mathrm{d}}{\mathrm{d}t}\mathbb{E}\left[S_2(t)\right] &= -2\theta\mathbb{E}\left[S_2(t)\right] + \sigma_Z^2\mathbb{E}\left[M_1(t)\right] - c_k\frac{\mathbb{E}\left[S_2(t)\right]^2}{\mathbb{E}\left[M_1(t)\right]}.
\end{aligned} \tag{22}$$

In order to find an expression at stationarity, we set the l.h.s. to zero and solve for M_1 and S_2.

$$\begin{aligned}
M_1^\infty &= \mu \\
S_2^\infty &= \frac{\sqrt{\mu^2(c_k\sigma_Z^2 + \theta^2)} - \mu\theta}{c_k}.
\end{aligned} \tag{23}$$

Hence, the suppressed and effective noise terms of $Z(t)$ at stationarity are given by

$$\begin{aligned}\mathbb{E}\left[\mathrm{Var}\left[Z(t) \mid \mathbf{X}_t\right]\right] &= \frac{\sqrt{\mu^2(c_k\sigma_Z^2+\theta^2)}-\mu\theta}{c_k} \\ \mathrm{Var}\left[\mathbb{E}\left[Z(t) \mid \mathbf{X}_t\right]\right] &= \mathrm{Var}\left[Z(t)\right] - \frac{\sqrt{\mu^2(c_k\sigma_Z^2+\theta^2)}-\mu\theta}{c_k}.\end{aligned} \tag{24}$$

Furthermore, the relative effective noise is found be dividing the effective noise by the total noise, i.e.,

$$\frac{\mathrm{Var}\left[\mathbb{E}\left[Z(t) \mid \mathbf{X}_t\right]\right]}{\mathrm{Var}\left[Z(t)\right]} = 1 + 2\frac{v^2}{c_k}\left(1-\sqrt{\frac{c_k}{v^2}+1}\right), \tag{25}$$

with $v = \theta/\sigma_Z$ as the normalized timescale of $Z(t)$.

S.5 Generalized master equations

Since the uncoupled process is non-Markovian, it does not satisfy a conventional master equation. Nevertheless, it can be described by a non-Markovian modification thereof, giving rise to a *generalized* master equation (GME). Typically, a GME is given in the form of an integro-differential equation, where the integral part stems from a time-convolution representing the *memory effects* of the system[1]. In some cases, such master equations may be given in a time-convolutionless form [8], which are typically more convenient to handle in practice. While GMEs are barely used in the context of biology, they are frequently applied in the field of quantum- and statistical mechanics [8, 10]. In the following we will use several concepts from that field to formulate a GME for the uncoupled dynamics. While a fully general description is yet to be developed, we consider the case where non-Markovian effects arise due to non-exponential and possibly time-dependent waiting-time distributions. However, the latter do not explicitly depend on the entire process history $\mathbf{X}_t$. This covers several relevant scenarios such as the master equation associated with the SNA.

Let us consider waiting-times $W_1, \ldots, W_N$, each of which is associated with a particular reaction channel. We assume that only the k-th reaction is modulated by the environmental network Z. Accordingly, only the k-th reaction will be associated with a non-exponential waiting-time distribution $P(W_k < w \mid \mathbf{X}_t) = P(W_k < w \mid X(t) = x)$ and corresponding density $p_k(w \mid x, t)$. For all other reaction channels we assume exponential waiting-time distributions $P(W_i < w \mid X(t) = x) = 1 - \exp(-h_i(x, c_i)w)$ and associated densities $p_i(w \mid x) = h_i(x, c_i)\exp(-h_i(x, c_i)w)$ for $i \neq k$. In the general case, the k-th waiting-time distribution follows

$$P(W_k < w \mid \mathbf{X}_t) = 1 - e^{-\int_0^w \lambda(\mathbf{X}^-_{t+T})\mathrm{d}T}, \tag{26}$$

with $\lambda(\mathbf{X}^-_{t+w}) = c_i\mathbb{E}\left[Z(t+w) \mid \mathbf{X}^-_{t+w}\right]$, where $\mathbf{X}^-_{t+w}$ extends $\mathbf{X}_t$ by a time-interval w assuming that no reaction of type k happens in w. The corresponding density is given by

$$p_k(w \mid \mathbf{X}_t) = \lambda(\mathbf{X}^-_{t+w})e^{-\int_0^w \lambda(\mathbf{X}^-_{t+T})\mathrm{d}T}. \tag{27}$$

Restricting the waiting-time distribution to only depend on the current state $X(t) = x$ and time t, we further have that

$$p_k(w \mid x, t) = \lambda(x, t+w)e^{-\int_0^w \lambda(x, t+T)\mathrm{d}T}. \tag{28}$$

Assuming that the process is in state x at time t, the next reaction and its firing time are determined by picking the minimum of the randomly drawn waiting-times $W_1, \ldots, W_N$, i.e.,

$$\begin{aligned} J &= \underset{i=1,\ldots,N}{\mathrm{argmin}}\; W_i \\ \text{and} \quad W &= W_j, \end{aligned} \tag{29}$$

[1]In contrast, Markovian dynamics are known to be memoryless.

with W as the waiting-time until the next reaction fires. Accordingly, we can define the probability that the next reaction will fire between t and $t+w$ as

$$\begin{aligned} P(W < w \mid X(t) = x) &= P(\min(W_1, \dots, W_N) < w \mid X(t) = x) \\ &= 1 - P(\min(W_1, \dots, W_N) > w \mid X(t) = x) \\ &= 1 - \prod_{i=1}^{N} P(W_i > w \mid X(t) = x). \end{aligned} \tag{30}$$

and the corresponding density will be denoted by $p(w \mid x, t)$. We remark that if all N waiting-times stem from an exponential distribution (i.e., if X is Markovian), also their minimum will be exponentially distributed. The probability of the next reaction at time w to be of type k is given by

$$\begin{aligned} Q_j(x, t, w) &= P(X(t+w) = x + \nu_j \mid X(t) = x, W = w) \\ &= \frac{P(W_j \in [w + \mathrm{d}w) \mid X(t) = x) \prod_{i \neq j} P(W_i > w \mid X(t) = x)}{P(W \in [w + \mathrm{d}w) \mid X(t) = x)} \\ &= \frac{p_j(w \mid x, t) \prod_{i \neq j} P(W_i > w \mid X(t) = x)}{p(w \mid x, t)}, \end{aligned} \tag{31}$$

where $p_j(w \mid x, t) = p_j(w \mid x)$ if $j \neq k$. Plugging in the waiting-time distribution from (28), we obtain transition probabilities

$$Q_i(x, t, w) = \begin{cases} \frac{\lambda(x, t+w)}{h(x, t+w)} & i = k \\ \frac{h_i(x, c_i)}{h(x, t+w)} & i \neq k \end{cases} \tag{32}$$

with $h(x,t) = \lambda(x,t) + \sum_{l \neq k} h_l(x, c_l)$ and the waiting-time density becomes

$$p(w \mid x, t) = h(x, t+w) e^{-\int_0^w h(x, t+T) \mathrm{d}T}. \tag{33}$$

The exponential term $e^{-\int_0^w h(x,t+T)\mathrm{d}T} = 1 - P(W < w \mid X(t) = x, t) := S(w \mid x, t)$ in (33) corresponds to the probability that the system remains in state x until $t + w$. Note that the transition probabilities depend on t and w only through their sum such that $Q_i(x, t, w) = Q_i(x, t + w)$.

Now, as similarly described in [5] or [6], we can use the above definitions to formulate a generalization of the Chapman-Kolmogorov equation for jump processes with non-exponential waiting-times. The probability of finding the system in a particular state x at time t is determined by considering two cases. If $x \neq X(0) = x_0$, we know that we have reached x from another state $x - \nu_i$ via a reaction of type i. The corresponding probability is obtained by summing over all possible reactions that may have moved the system to a state x and all possible times at which the reaction may have fired. The latter will be reflected by the aforementioned time-integral. A special case arises if $x = x_0$, since we need an additional term accounting for the case where $X(t)$ has never changed from its initial condition. In summary, one can show that the probability $P(x, t)$ satisfies

$$\begin{aligned} P(x,t) &= \underbrace{S(t \mid x, 0) \mathbb{1}_{x_0}(x)}_{\text{No reaction until } t.} + \underbrace{\sum_{i=1}^{N} \int_0^t P(x - \nu_i, t - w) Q_i(x, t - w) h(x - \nu_i, t - w) S(w \mid x, t - w) \mathrm{d}w}_{\text{Arrived in } x \text{ through reaction } i.} \\ &= S(t \mid x, 0) \mathbb{1}_{x_0}(x) + \sum_{i=1}^{N} \int_0^t P(x - \nu_i, t - w) h_i(x - \nu_i, t - w) S(w \mid x, t - w) \mathrm{d}w. \end{aligned} \tag{34}$$

Eq. (34) can be rewritten in differential form as

$$\begin{aligned} \frac{\mathrm{d}}{\mathrm{d}t} P(x,t) = \sum_{i=1}^{N} \int_0^t \Big(& P(x - \nu_i, t - w) Q_i(x - \nu_i, t - w) \phi(w \mid x, t - w) \\ & - P(x, t - w) Q_i(x, t - w) \phi(w \mid x, t - w) \Big) \mathrm{d}w, \end{aligned} \tag{35}$$

where the *memory function* $\phi(w \mid x,t)$ can in principle be derived from the waiting-time distribution $p(w \mid x,t)$. However, we are not aware of a fully general relation but if $p(w \mid x,t) = p(w \mid x)$, i.e., it does *not* explicitly depend on time, $\phi(w \mid x,t)$ is given by the *Montroll-Weiss* equation (ref. [27] in the main text)

$$\phi(u) = \frac{up(u)}{1-p(u)}, \tag{36}$$

where $\phi(u)$ and $p(u)$ denote the Laplace transforms of $p(w \mid x)$ and $\phi(w \mid x)$, respectively. From this relation, it is straight-forward to verify that the master equation becomes memory-less in case of exponential waiting-time distributions (i.e., the memory function is given by a dirac-delta function) [5, 6].

For the case considered here, we derive a master equation by direct differentiation of the Chapman-Kolmogorov equation (i.e., similar to [7]). With the above definitions, (34) becomes

$$\begin{aligned} P(x,t) &= e^{-\int_0^t h(x,T)\mathrm{d}T}\mathbb{1}_{x_0}(x) + \sum_{i=1}^N \int_0^t P(x-\nu_i, t-w)h_i(x-\nu_i, t-w)e^{-\int_0^w h(x,t-w+T)\mathrm{d}T}\mathrm{d}w \\ &= e^{-\int_0^t h(x,T)\mathrm{d}T}\mathbb{1}_{x_0}(x) + \sum_{i=1}^N \int_0^t P(x-\nu_i, w)h_i(x-\nu_i, w)e^{-\int_0^{t-w} h(x,w+T)\mathrm{d}T}\mathrm{d}w \\ &= e^{-\int_0^t h(x,T)\mathrm{d}T}\left[\mathbb{1}_{x_0}(x) + \sum_{i=1}^N \int_0^t P(x-\nu_i, w)h_i(x-\nu_i, w)e^{\int_0^w h(x,T)\mathrm{d}T}\mathrm{d}w\right] \end{aligned} \tag{37}$$

and taking the time-derivative further yields

$$\begin{aligned} \frac{\mathrm{d}}{\mathrm{d}t}P(x,t) &= e^{-\int_0^t h(x,T)\mathrm{d}T}\sum_{i=1}^N P(x-\nu_i,t)h_i(x-\nu_i,t)e^{\int_0^t h(x,T)\mathrm{d}T} \\ &\quad - h(x,t)\underbrace{e^{-\int_0^t h(x,T)\mathrm{d}T}\left[\mathbb{1}_{x_0}(x) + \sum_{i=1}^N \int_0^t P(x-\nu_i, w)h_i(x-\nu_i, w)e^{\int_0^w h(x,T)\mathrm{d}T}\mathrm{d}w\right]}_{P(x,t)}. \end{aligned} \tag{38}$$

With $h(x,t) = \lambda(x,t) + \sum_{l\neq k} h_l(x,c_l)$ we arrive at

$$\frac{\mathrm{d}}{\mathrm{d}t}P(x,t) = \sum_{i\neq k}^N \Big[h_i(x-\nu_i,c_l)P(x-\nu_i,t) - h_i(x,c_i)P(x,t)\Big] + \lambda(x-\nu_k,t)P(x-\nu_k,t) - \lambda(x,t)P(x,t). \tag{39}$$

Eq.(39) illustrate that under the given assumptions, the uncoupled dynamics can be described by a non-stationary and convolution-less master equation.

S.5.1 Derivation of the slow noise approximation

We apply the time-convolutionless master equation approach in order to approximate the transient probability distribution of a birth-death process in a fluctuating environment, i.e.,

$$\emptyset \xrightarrow{Z(t)} X \xrightarrow{c_d} \emptyset,$$

where the environment Z enters the model through its birth-rate and c_d is the rate constant of the death-reaction. The birth-hazard of the uncoupled process X is then given by

$$\lambda(\mathbf{X}_t) = \mathbb{E}\left[Z(t) \mid \mathbf{X}_t\right]. \tag{40}$$

The slow noise approximation is based on two critical assumptions:

1. The conditional process $Z(t) \mid \mathbf{X}_t$ can be well represented by a Gamma distribution with time-varying parameters. We highlight that this assumption does *not* mean that the unconditional process $Z(t)$ needs to be approximately Gamma-distributed.
2. The impact of a fluctuating environment on a system can be well "mimicked" by a static environment $\bar{Z}$ with a suitably chosen variance (e.g., the effective noise).

Under the Gamma-assumption, the conditional expectation from (40) is governed by the differential equations

$$\begin{aligned} \mathrm{d}M_1 &= \left[\mathcal{D}_1 - (M_2 - M_1^2)\right] \mathrm{d}t + \frac{M_2 - M_1^2}{M_1} \mathrm{d}R_b \\ \mathrm{d}M_2 &= \left[\mathcal{D}_2 - 2\frac{M_2}{M_1}\left(M_2 - M_1^2\right)\right] \mathrm{d}t + 2\left[\frac{M_2^2}{M_1^2} - M_2\right] \mathrm{d}R_b. \end{aligned} \tag{41}$$

Assuming a very slow environmental dynamics, we have that $\mathcal{D}_1$ and $\mathcal{D}_1$ become zero and hence,

$$\begin{aligned} \mathrm{d}M_1 &= -(M_2 - M_1^2)\mathrm{d}t + \frac{M_2 - M_1^2}{M_1} \mathrm{d}R_b \\ \mathrm{d}M_2 &= -2\frac{M_2}{M_1}\left(M_2 - M_1^2\right) \mathrm{d}t + 2\left[\frac{M_2^2}{M_1^2} - M_2\right] \mathrm{d}R_b. \end{aligned} \tag{42}$$

The solution of (42) immediately before the next jump at time t is given by

$$\begin{aligned} M_1^-(t) &= \frac{M_1^2(0)}{M_1(0) + (M_2(0) - M_1^2(0))\, t} \\ M_2^-(t) &= \frac{M_1^2(0) M_2(0)}{\left[M_1(0) + (M_2(0) - M_1^2(0))\, t\right]^2}. \end{aligned} \tag{43}$$

Adding the the jump term $\left[M_2 - M_1^2\right]/M_1$ to $M_1^-(t)$ further yields

$$M_1^+(t) = \frac{M_1^2(0) + \left(M_2(0) - M_1^2(0)\right)}{M_1(0) + (M_2(0) - M_1^2(0))\, t}. \tag{44}$$

Repeating the above procedure for the subsequent jumps, we obtain

$$\begin{aligned} M_1^+(t) &= \frac{M_1^2(0) + \left(M_2(0) - M_1^2(0)\right) R_b(t)}{M_1(0) + (M_2(0) - M_1^2(0))\, t} \\ &= \frac{\mu^2}{\mu + \sigma^2 t} + \frac{\sigma^2}{\mu + \sigma^2 t} R_b(t) \\ &= \lambda(R_b(t), t), \end{aligned} \tag{45}$$

with $\mu = \mathbb{E}\left[\bar{Z}\right]$ and $\sigma^2 = \mathrm{Var}\left[\bar{Z}\right]$ as mean and variance of the approximate environment $\bar{Z}$. Importantly, we find that the conditional mean – and therefore the hazard function only depends on the number of birth reactions but not on when those reactions happened. The uncoupled dynamics can therefore be described by eq. (39). Using the fact that $X(t) = R_b(t) - R_d(t)$ for $X(0) = 0$, we obtain the time-convolutionless master equation

$$\begin{aligned} \frac{\mathrm{d}}{\mathrm{d}t} P(r_b, r_d, t) = \lambda(r_b - 1, t) P(r_b - 1, r_d, t) + c_d \left[r_b - r_d + 1\right] P(r_b, r_d - 1, t) \\ - \lambda(r_b, t) P(r_b, r_d, t) - c_d \left[r_b - r_d\right] P(r_b, r_d, t). \end{aligned} \tag{46}$$

In the following, we derive a solution of this equation using the concept of generating functions [3]. In particular, we employ certain properties of the probability generating function

$$\gamma(\eta_b, \eta_d, t) = \sum_{r_d=0}^{\infty} \sum_{r_b=0}^{\infty} \eta_b^{r_b} \eta_d^{r_d} p(r_b, r_d, t), \tag{47}$$

which allow us to transform the difference-differential equation (46) into a PDE. It is straight-forward to show that the discrete shifts in $P(r_b, r_d, t)$ map to partial derivates of the probability generating function yielding

$$\begin{aligned}\frac{\partial}{\partial t}\gamma(\eta_b,\eta_d,t) = {} & \frac{\mu^2}{\mu+\sigma^2 t}\left(\eta_b-1\right)\gamma(\eta_b,\eta_d,t) + \eta_b\Big[\frac{\sigma^2}{\mu+\sigma^2 t}\left(\eta_b-1\right) + c_d\left(1-\eta_d\right)\Big]\frac{\partial}{\partial \eta_b}\gamma(\eta_b,\eta_d,t) \\ & + c_d\eta_d\left(\eta_d-1\right)\frac{\partial}{\partial \eta_d}\gamma(\eta_b,\eta_d,t).\end{aligned} \tag{48}$$

We realize that the above equation is a linear PDE with time-varying coefficients, which we aim to solve using the method of characteristics. This method is based on describing the PDE by means of so-called *characteristic curves* that are given through a set of coupled ODEs – each of them corresponding to a particular dimension of the PDE (i.e., η_b, η_d and γ). Considering a general linear first-order PDE with three independent variables, i.e.,

$$\frac{\partial}{\partial t}\gamma(\eta_b,\eta_d,t) = a(\eta_b,\eta_d,t,\gamma)\frac{\partial}{\partial \eta_b}\gamma(\eta_b,\eta_d,t) + b(\eta_b,\eta_d,t,\gamma)\frac{\partial}{\partial \eta_d}\gamma(\eta_b,\eta_d,t) + c(\eta_b,\eta_d,t,\gamma) \tag{49}$$

the characteristic equations are given by

$$\frac{\mathrm{d}}{\mathrm{d}t}\eta_b(t) = -a(\eta_b(t),\eta_d(t),t,\gamma(t)) \tag{50}$$

$$\frac{\mathrm{d}}{\mathrm{d}t}\eta_d(t) = -b(\eta_b(t),\eta_d(t),t,\gamma(t)) \tag{51}$$

$$\frac{\mathrm{d}}{\mathrm{d}t}\gamma(t) = c(\eta_b(t),\eta_d(t),t,\gamma(t)). \tag{52}$$

In the special case of eq. (48), we have that

$$\frac{\mathrm{d}}{\mathrm{d}t}\eta_b(t) = -\eta_b(t)\left(\eta_b(t)\frac{\sigma^2}{\mu+\sigma^2 t} - c_d\eta_d(t) - \frac{\sigma^2}{\mu+\sigma^2 t} + c_d\right) \tag{53}$$

$$\frac{\mathrm{d}}{\mathrm{d}t}\eta_d(t) = -c_d\eta_d(t)(\eta_d(t)-1) \tag{54}$$

$$\frac{\mathrm{d}}{\mathrm{d}t}\gamma(t) = \frac{\mu^2}{\mu+\sigma^2 t}\gamma(t)(\eta_b(t)-1), \tag{55}$$

whose solution is given by

$$\eta_b(t) = \frac{Ac_d\left(Be^{c_d t}-B+1\right)\left(\mu+\sigma^2 t\right)}{-AB\sigma^2 e^{c_d t} + ABc_d\sigma^2 t e^{c_d t} + AB\sigma^2 + A\sigma^2 e^{c_d t} - A\sigma^2 + c_d\mu e^{c_d t}} \tag{56}$$

$$\eta_d(t) = \frac{Be^{c_d t}}{Be^{c_d t}-B+1} \tag{57}$$

$$\gamma(t) = e^{-\frac{c_d\mu^2 t}{\sigma^2}}\left(\frac{e^{c_d t}\left(c_d\left(AB\sigma^2 t+\mu\right) - A(B-1)\sigma^2\right) + A(B-1)\sigma^2}{c_d\left(\mu+\sigma^2 t\right)}\right)^{\frac{\mu^2}{\sigma^2}}, \tag{58}$$

with $\eta_b(0) = A$, $\eta_d(0) = B$ and $\gamma(0) = 1$. In order the obtain the general solution of γ, we need to express the initial conditions A and B as functions of η_b and η_d using (56) and (57). The probability generating function γ is finally given by

$$\gamma(\eta_b,\eta_d,t) = \left(\frac{c_d\mu}{c_d\left(\mu+\sigma^2 t(1-\eta_b\eta_d)\right) + \sigma^2\eta_b(\eta_d-1)\left(e^{c_d t}-1\right)}\right)^{\frac{\mu^2}{\sigma^2}}. \tag{59}$$

Back-transformation then yields the joint probability distribution over r_b and r_d, i.e.,

$$\begin{aligned}P(r_b,r_d,t) &= \frac{1}{r_b! r_d!}\frac{\partial^{r_b+r_d}}{\partial\eta_b^{r_b}\partial\eta_d^{r_d}}\gamma(\eta_b,\eta_d,t)\Bigg|_{\eta_b=0,\eta_d=0} \\ &= \frac{\left(\frac{\mu}{\mu+\sigma^2 t}\right)^{\frac{\mu^2}{\sigma^2}}\Gamma\left(\frac{\mu^2}{\sigma^2}+r_b\right)}{r_d!(r_b-r_d)!\Gamma\left(\frac{\mu^2}{\sigma^2}\right)}\left(\frac{\sigma^2\left(e^{c_d t}-1\right)}{c_d\left(\mu+\sigma^2 t\right)}\right)^{r_b}\left(\frac{c_d t - e^{c_d t}+1}{e^{c_d t}-1}\right)^{r_d},\end{aligned} \tag{60}$$

from which we compute the distribution in X as

$$\begin{aligned} P(x,t) &= \sum_{r_b=x}^{\infty} P(r_b, r_b - x, t) \\ &= \mathcal{NB}\left(x; \frac{\mu^2}{\sigma^2}, \frac{c_d \mu e^{c_d t}}{c_d \mu e^{c_d t} + \left(e^{c_d t} - 1\right)\sigma^2}\right), \end{aligned} \tag{61}$$

i.e., a negative binomial distribution. Furthermore, it is straight-forward to show that marginally, both r_b and r_d have negative binomial distributions. We remark that the slow noise approximation is exact in the case of infinitely slow as well as fast fluctuations.

References

[1] O. Aalen. Nonparametric inference for a family of counting processes. *Ann Stat*, 6(4):701–726, 1978.

[2] O. Aalen. Mixing distributions on a Markov chain. *Scand J Statist*, 14(4):281–289, 1987.

[3] C. W. Gardiner et al. *Handbook of stochastic methods*, volume 3. Springer Berlin, 1985.

[4] A. Gelman, J. B. Carlin, H. S. Stern, D. B. Dunson, A. Vehtari, and D. B. Rubin. *Bayesian data analysis*. CRC press, 2013.

[5] D. Gillespie. Master equations for random walks with arbitrary pausing time distributions. *Physics Letters A*, 64(1):22–24, 1977.

[6] V. Kenkre, E. Montroll, and M. Shlesinger. Generalized master equations for continuous-time random walks. *Journal of Statistical Physics*, 9(1):45–50, 1973.

[7] J. R. Norris. *Markov chains*. Number 2008. Cambridge university press, 1998.

[8] F. Shibata, Y. Takahashi, and N. Hashitsume. A generalized stochastic Liouville equation. non-Markovian versus memoryless master equations. *Journal of Statistical Physics*, 17(4):171–187, 1977.

[9] C. Zechner, S. Deb, and H. Koeppl. Marginal dynamics of stochastic biochemical networks in random environments. *2013 European Control Conference (ECC)*, pages 4269–4274, 2013.

[10] R. Zwanzig. On the identity of three generalized master equations. *Physica*, 30(6):1109–1123, 1964.
